# Achieving Tunable Cold/Warm White-Light Emission in a Single Perovskite Material with Near-Unity Photoluminescence Quantum Yield

**DOI:** 10.1007/s40820-023-01168-5

**Published:** 2023-08-31

**Authors:** Bo Zhou, Aixuan Du, Dong Ding, Zexiang Liu, Ye Wang, Haizhe Zhong, Henan Li, Hanlin Hu, Yumeng Shi

**Affiliations:** 1 International Collaborative Laboratory of 2D Materials for Optoelectronics Science and Technology of Ministry of Education, Institute of Microscale Optoelectronics, Shenzhen University, Shenzhen, 518060 People’s Republic of China; 2 Shandong Laboratory of Yantai Advanced Materials and Green Manufacturing, Yantai, 264006 People’s Republic of China; 3 Key Laboratory of Material Physics, School of Physics and Microelectronics, Zhengzhou University, Ministry of Education, Zhengzhou, 450052 People’s Republic of China; 4 School of Electronics and Information Engineering, Shenzhen University, Shenzhen, 518060 People’s Republic of China; 5 Hoffmann Institute of Advanced Materials, Shenzhen Polytechnic, Shenzhen, 518060 People’s Republic of China

**Keywords:** 0D perovskite, Multi-ion doping, Near-unity white light, Energy transfer, Self-trapped excitons

## Abstract

Single materials that exhibit efficient and stable white-light emission are highly desirable for lighting applications. This paper reports a novel zero-dimensional perovskite, Rb_4_CdCl_6_:Sn^*2*+^_,_ Mn^*2*+^, which demonstrates exceptional white-light properties including adjustable correlated color temperature, high color rendering index of up to 85, and near-unity photoluminescence quantum yield of 99%. Using a co-doping strategy involving Sn^2+^ and Mn^2+^, cyan-orange dual-band emission with complementary spectral ranges is activated by the self-trapped excitons and *d-d* transitions of the Sn^2+^ and Mn^2+^ centers in the Rb_4_CdCl_6_ host, respectively. Intriguingly, although Mn^2+^ ions doped in Rb_4_CdCl_6_ are difficult to excite, efficient Mn^2+^ emission can be realized through an ultra-high-efficient energy transfer between Sn^2+^ and Mn^2+^ via the formation of adjacent exchange-coupled Sn–Mn pairs. Benefiting from this efficient Dexter energy transfer process, the dual emission shares the same optimal excitation wavelengths of the Sn^2+^ centers and suppresses the non-radiative vibration relaxation significantly. Moreover, the relative intensities of the dual-emission components can be modulated flexibly by adjusting the fraction of the Sn^2+^ ions to the Sn–Mn pairs. This co-doping approach involving short-range energy transfer represents a promising avenue for achieving high-quality white light within a single material.

**Supplementary Information:**

The online version contains supplementary material available at 10.1007/s40820-023-01168-5.

## Highlights

- High-quality Sn^2+^/Mn^2+^-co-doped Rb_4_CdCl_6_ single crystals and powders were prepared and showed high-performance dual-emission white light with near-unity photoluminescence quantum yield.
- Short-range and extremely strong interactions between Sn^2+^ and Mn^2+^ were observed that lead to an intriguing ultra-high-efficiency Dexter energy transfer process from adjacent Sn^2+^ ions to Mn^2+^ ions.
- The dual-emission intensities were tuned flexibly by varying the fractions of Sn^2+^ and Sn–Mn pairs to balance their emission proportions for cold/warm white-light generation.

**Supplementary Information:**

The online version contains supplementary material available at 10.1007/s40820-023-01168-5.

## Graphical abstract

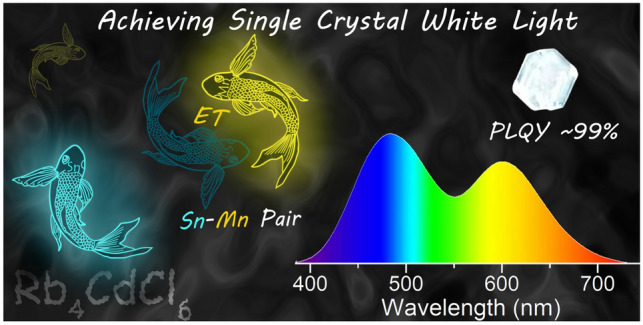

## Introduction

Metal halide perovskites, specifically those containing self-trapped excitons (STEs), have garnered significant attention as promising candidates for white-light-emitting diodes (WLEDs) because of their ultra-broadband emission characteristics and high photoluminescence quantum yields (PLQYs) [1–9]. The ability to emit light across the entire visible spectrum enables the realization of white light from a single emitter layer, simplifying device fabrication [3, 10]. While achieving high efficiency is crucial, it is equally important to attain a high color render index (CRI) and adjustable correlated color temperature (CCT) for applications that require high-quality and color-critical lighting, such as art galleries, photography, surgery, and military signs [11–13]. However, meeting these requirements using a single broadband emission source poses significant challenges [14].

Integrating auxiliary emission channels or components with different emission wavelengths into the broadband emission is an effective strategy to improve CRI and adjust CCT [15, 16]. While the assembly of multi-band emissions from chemically similar and coexisting perovskite materials has led to the successful realization of high-quality white light, coordinating different emissions within a single material offers distinct advantages. This design approach avoids color instability caused by different degradation rates of the emitters and minimizes efficiency losses due to emission reabsorption [17]. Generally, in metal halide perovskite materials, the [BX_6_]^n−^ octahedra serve as the fundamental functional units, and the interactions between these units dictate the material’s physical properties [18, 19]. Leveraging the structural tolerance of perovskite materials, different kinds of [BX_6_]^n−^ octahedra can be assembled in a single material to achieve various optical properties [20–23]. Recently, the integration of hybrid B-site metals has enabled the realization of multiple emission channels with individually tuned wavelengths and the achievement of high-quality white-light within a single perovskite material [24–29].

All-inorganic perovskites offer advantages in terms of stability against heat and UV radiation compared to hybrid organic–inorganic perovskites, which are prone to photobleaching and aging of organic functional groups [3, 30]. However, the complex interactions between identical and different [BX_6_]^n−^ octahedra within a compact lattice should be carefully considered. For example, all-inorganic perovskites often exhibit low intrinsic PL intensity due to the indirect bandgap and significant concentration quenching [31]. Achieving efficient broadband STEs emission requires appropriate dilution of n*s*^2^ ions (i.e., ions that have outermost *s*^2^ electrons, such as Pb^2+^, Sn^2+^, Sb^3+^, Bi^3+^, Te^4+^) into a host material with large bandgap [32]. Incorporation of additional optical dopant centers to generate auxiliary emissions without significant quenching of the overall PLQY remains a challenge [17]. Successful achievement of efficient and tunable white light in a single all-inorganic perovskite has been demonstrated in 2021, such as co-doping Sb^3+^ and Bi^3+^ in Cs_2_NaInCl_6_ [24]. Over the years, various combinations of optical dopants have been developed, including Sb^3+^/Bi^3+^-co-doped Cs_2_SnCl_6_ [25], Bi^3+^/Te^4+^-co-doped Cs_2_SnCl_6_ [26, 27], and Sb^3+^-doped Cs_2_ZrCl_6_ [28, 29]. However, these different emission sources often require distinct optimal excitation ranges. Finding a common excitation wavelength that aligns with the optimal excitations of each emission source remains challenging, leading to unstable color rendering and significant efficiency losses.

Energy-transfer-related fluorescence between n*s*^2^ ion sensitizers and Ln^3+^/TM^n+^ (Ln: lanthanides, TM: transition metals) ion activators enable the emission from TM^n+^ centers to possess the same excitation profile as STEs emission from the n*s*^2^ centers [33]. In our previous work, we incorporated rare-earth Er^3+^ and Ho^3+^ ions with Sb^3+^ in Cs_2_NaInCl_6_ host, demonstrating the feasibility of achieving tunable white-light via an energy transfer design [34]. The Er^3+^, Ho^3+^ optical centers exhibited sharp-band green and red emission sensitized by Sb^3+^, with an identical excitation profile to the primary broadband blue emission. While the sharp-band emission from rare earths efficiently adjusted the CCT, they contributed minimally to the improvement of CRI. Mn^2+^ ions were considered as a more suitable alternative to co-doped with n*s*^2^ ions compared to Ln^3+^ ions, owing to the broadband emission from *d-d* transition [35]. Despite the ease of constructing energy transfer between n*s*^2^ ions and Mn^2+^, achieving efficient white light has remained a challenge [36–39]. Dexter energy transfer is short-range interaction which moves an exciton (i.e., an electron–hole pair) from a donor to an acceptor [40]. The free excitons generated by n*s*^2^ ions in the metal halides lattice upon photon absorption possess the potential to form DET pairs with Ln^3+^/TM^n+^ ions and thereby enhance the optical properties. However, the Dexter energy transfer has rarely been observed in metal halides.

In this work, we selected the broadband cyan STEs emission in Sn^2+^-doped Rb_4_CdCl_6_ as the primary emission. Through further co-doping of Mn^2+^, a typical short-range Dexter energy transfer from Sn^2+^ to Mn^2+^ was generated, which not only produced an additional orange broadband emission for high-quality dual-emission white light but also enhanced the total PLQY to near-unity. The dual-emission shows almost the same excitation spectra originate from Sn^2+^ ions, and their relative intensities can be continuously tuned by the fractions of Sn^2+^ and Sn–Mn pairs to balance their emission proportions for cold/warm white-light generation.

## Experimental Section

### Materials

RbCl (99.99% metals basis), CdCl_2_ (anhydrous, 99.99% metals basis), SnCl_2_·2H_2_O (99.99% metals basis), hypophosphorous acid (H_3_PO_2_, 50 wt% in H_2_O), isopropanol (IPA, AR), and dimethylformamide (DMF, AR) were purchased from Aladdin. MnCl_2_·4H_2_O (99% metals basis) was purchased from Alfa Aesar. Hydrochloric acid (HCl, AR) was purchased from Guangzhou Chemical Reagent Factory. All chemicals were used as received without any further purification.

### Preparation of Single Crystal, Powder, and LEDs

#### ***Preparation of Sn***^***2***+^***, Mn***^***2***+^, ***and Sn***^***2***+^***/Mn***^***2***+^-***Co-Doped Rb***_***4***_***CdCl***_***6***_*** Powders***

1 mmol (0.121 g) of RbCl was dissolved in 1 mL of HCl to prepare a 1 M RbCl solution. 2 mmol (0.367 g) of CdCl_2_ was dissolved in 1 mL of HCl to prepare a 2 M CdCl_2_ solution. 0.1 mmol (0.023 g) of SnCl_2_·2H_2_O was dissolved in a mixture of 1 mL of HCl and 0.1 mL of H_3_PO_2_ to prepare a 0.1 M SnCl_2_ solution. 1 mmol (0.198 g) of MnCl_2_·4H_2_O was dissolved in 1 mL of HCl to prepare a 1 M MnCl_2_ solution. The CdCl_2_ solution (0.5 mL) was premixed with IPA (5 mL), and then, the RbCl solution (4 mL) was rapidly injected into the mixture to obtain pure Rb_4_CdCl_6_ precipitation. To obtain the Sn^2+^ (or Mn^2+^)-doped Rb_4_CdCl_6_ samples, SnCl_2_ (or MnCl_2_) solution should be added to the CdCl_2_ solution while maintaining a total amount of SnCl_2_ (or MnCl_2_) and CdCl_2_ of 1 mmol [M^II^/(M^II^ + Cd) × 100%]. All precipitations were dried at 50 °C for 30 min.

#### ***Growth of Sn***^***2***+^***/Mn***^***2***+^-***Co-Doped Rb***_***4***_***CdCl***_***6***_*** Single Crystals***

The synthesis of the Sn^2+^/Mn^2+^-co-doped Rb_4_CdCl_6_ single crystal was based on a hydrothermal method. First, 16 mmol of RbCl, 4 mmol of CdCl_2_, 0.8 mmol of SnCl_2_, and 0.8 mmol of MnCl_2_ were added to a 25-mL Teflon liner. Then, 8 mL of HCl, 8 mL of DMF, and 0.8 mL of H_3_PO_2_ were injected into the Teflon liner. The Teflon liner was sealed in a stainless-steel autoclave and then was heated to 120 °C for 10 h. The reaction mixture was subsequently cooled to room temperature at a rate of 3 °C h^−1^. Finally, the samples were cleaned with IPA and dried at 50 °C for 30 min.

#### Fabrication of Down-Conversion WLEDs

One gram of the Sn^2+^/Mn^2+^-co-doped Rb_4_CdCl_6_ powder sample was dispersed into 1 g of organic silicone, and then, the gel mixture obtained was cast on the surface of an LED chip (purchased from Shenzhen Ruibaoguang Technology Co., Ltd.) with an emission wavelength of 295 nm. Finally, the LEDs obtained by this method were dried at 80 °C for 24 h in a vacuum drying chamber.

### Characterization

Powder X-ray diffraction (XRD) measurements were taken using a D2 diffractometer (Bruker, Billerica, MA, USA) with Cu Kα radiation (*λ* = 1.5418 Å). Energy-dispersive X-ray spectroscopy (EDS) characterization of the microcrystal was performed using a field-emission scanning electron microscope (FESEM; SU8010, Hitachi). UV–visible absorption spectroscopy was performed using a UV–Vis–near-infrared (NIR) spectrometer (UV-2600i, Shimadzu, Kyoto, Japan). The photoluminescence (PL), photoluminescence excitation (PLE) spectra, and PLQY measurements were taken using a fluorescence spectrophotometer (FS5, Edinburgh Instruments, Livingston, UK). The time-resolved PL spectroscopy measurements were also taken using the FS5 fluorescence spectrometer with a microsecond lamp acting as its excitation source. Inductively coupled plasma optical emission spectroscopy (ICP-OES) was performed using an atomic emission spectrometer (JY2000-2, Horiba, Japan). The zero-field-cooled (ZFC) and field-cooled (FC) curve measurements were taken using an integrated physical property measurement system (PPMS-9 T, Quantum Design, USA). Electron paramagnetic resonance (EPR) characterization was performed using an electronic paramagnetic resonance spectrometer (EPR200-Plus, Chinainstru & Quantumtech (CIQTEK), China). The PL spectra of the LED for different drive currents were measured using a ScanPro Advance system (Metatest, China).

## Results and Discussion

**Fig. 1:**
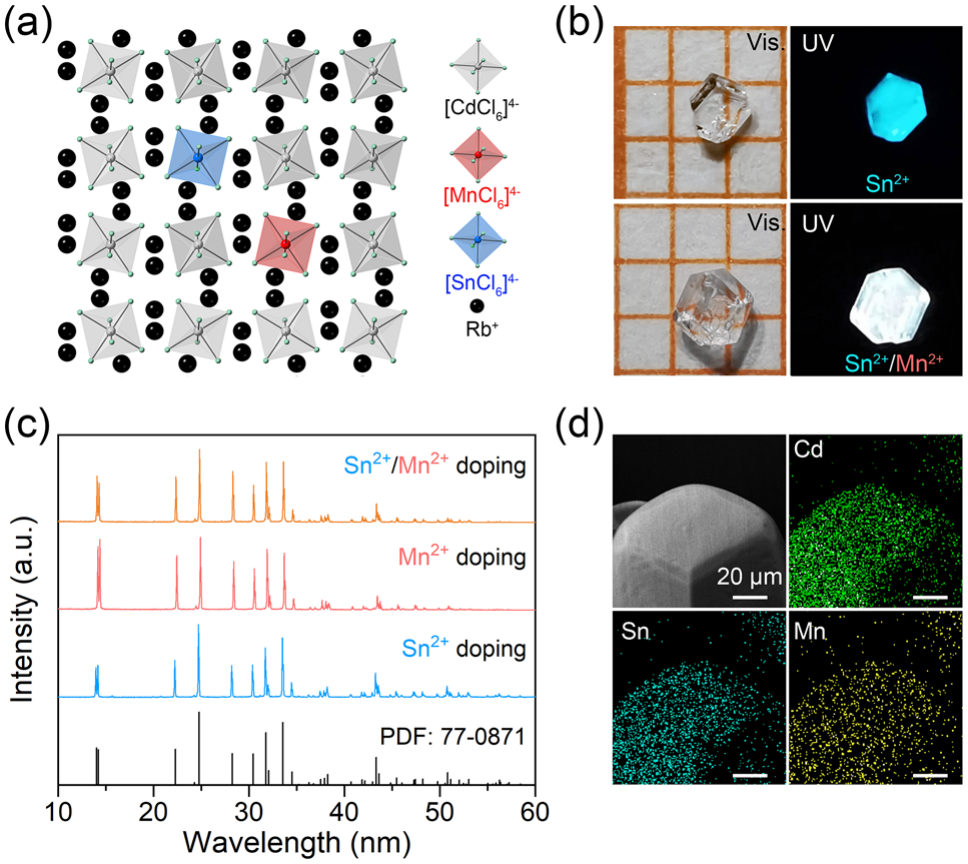
**a** Schematic diagram of Rb_4_CdCl_6_ crystal structure and substitutions performed via Sn^2+^ and Mn^2+^ co-doping. **b** Optical images of Sn^2+^-doped and Sn^2+^/Mn^2+^-co-doped single crystals under daylight and UV light illumination. **c** Powder XRD patterns of Sn^2+^, Mn^2+^, and Sn^2+^/Mn^2+^-co-doped Rb_4_CdCl_6_ samples; PDF 77-0871 is shown for comparison. **d** Energy-dispersive X-ray spectroscopy (EDS) mapping of the Sn^2+^/Mn^2+^-co-doped Rb_4_CdCl_6_ microcrystal

The crystal structure of Rb_4_CdCl_6_ is illustrated in Fig. 1a, exhibiting a characteristic zero-dimensional (0D) crystal structure with trigonal *R*-3c symmetry, resembling that of Cs_4_PbBr_6_ [41]. In this structure, the [CdCl_6_]^4−^ octahedra are completely isolated by Rb^+^ ions [42]. Substituting Cd^2+^ ions with Sn^2+^ and Mn^2+^ ions lead to the formation of [SnCl_6_]^4−^ and [MnCl_6_]^4−^ octahedra, respectively, as they have comparable radii and the same divalent charge as Cd^2+^. An optical image of a single crystal of Sn^2+^/Mn^2+^-co-doped Rb_4_CdCl_6_ prepared via a hydrothermal method is shown in Fig. 1b [43]. The crystal appears colorless under daylight, due to the extremely weak absorption of Mn^2+^ ions doping in Rb_4_CdCl_6_ (Fig. S1), which distinguishes it from most of Mn^2+^-doped/based perovskites [35, 44–46]. However, under UV light, the crystal emits bright white light, in contrast to the cyan emission of Sn^2+^-doped Rb_4_CdCl_6_ [43], confirming the successful incorporation Mn^2+^ ions into the Rb_4_CdCl_6_. Figure 1c shows the powder X-ray diffraction (PXRD) pattern of as-prepared Sn^2+^ and Mn^2+^-co-doped Rb_4_CdCl_6_ samples. The observed PXRD patterns match well with the standard powder diffraction file [PDF 77–0871]. It is worth noting that Sn^2+^ and Mn^2+^ ions have larger and smaller ion radii (*r* = 1.12 and 0.83 Å, coordination number (CN) = 6), respectively, compared to Cd^2+^ ions (*r* = 0.95 Å, CN = 6), and the co-doping of Sn^2+^ and Mn^2+^ would offset each other's influence on the cell volume. Scanning electron microscopy (SEM) with energy-dispersive spectral mapping (EDS mapping) was employed to analyze the samples. Figure 1d shows that Sn^2+^ and Mn^2+^ dopants are uniformly distributed in the microcrystal, confirming the successful preparation of the co-doped samples. Inductively coupled plasma optical emission spectroscopy (ICP-OES) was utilized to determine the doping ratio of Sn^2+^ and Mn^2+^ in the samples, considering both fixed and varying feed ratios of Sn^2+^ and Mn^2+^. As depicted in Fig. S2, the actual amount of Sn^2+^ is ~ 4.5% in the different samples, slightly lower than the 5% feeding ratio. The actual amount of Mn^2+^ also aligns with the feed ratio due to homovalent substitution.

**Fig. 2:**
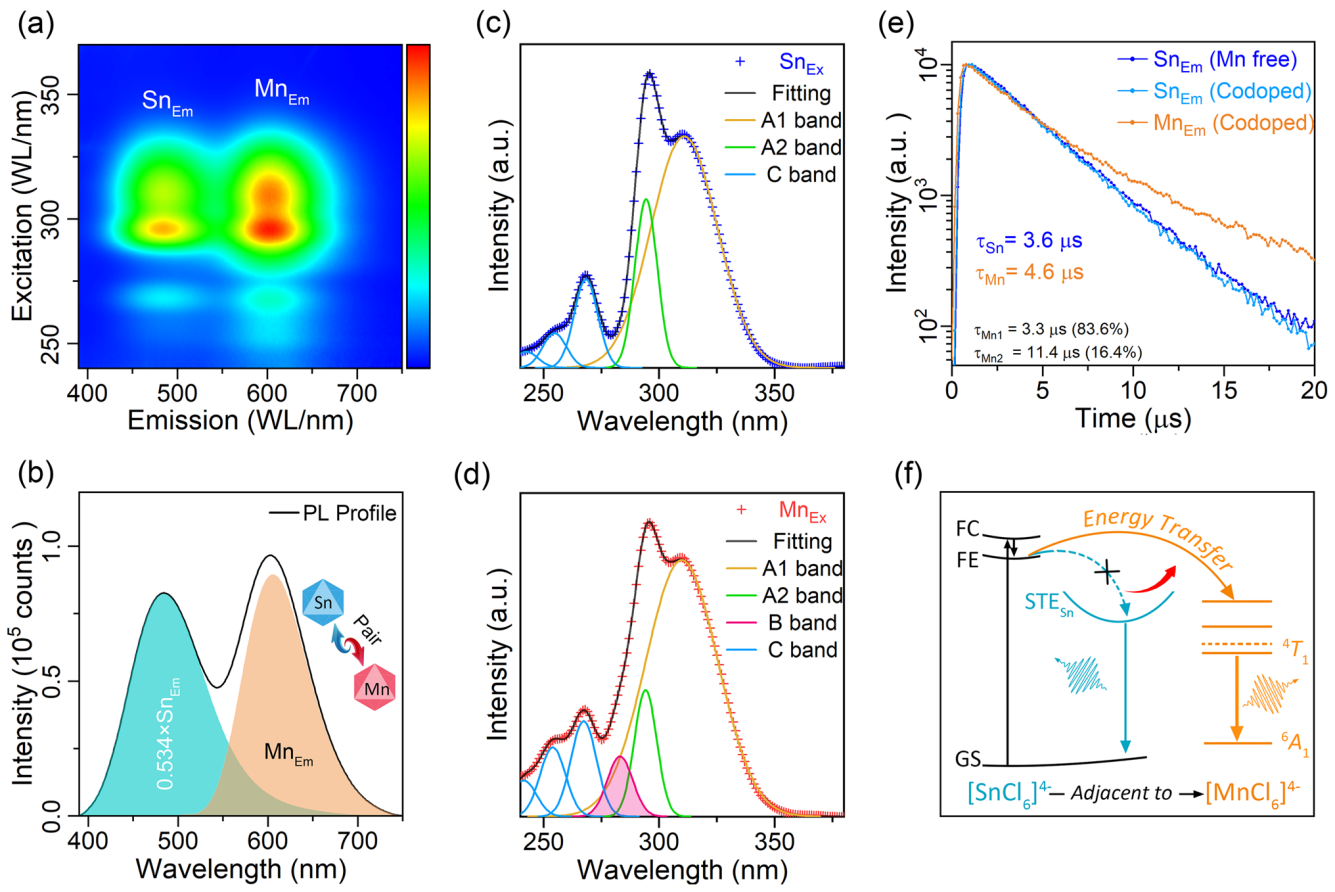
**a** Pseudo-color map of the PL/PLE of Sn^2+^/Mn^2+^-co-doped Rb_4_CdCl_6_. **b** Dual-emission profile formed as a combination of two components, Sn_Em_ and Mn_Em_. **c**, **d** Excitation profiles corresponding to Sn_Em_ and Mn_Em_, respectively, consisting of A, B, and C bands. **e** Decay curves obtained for Sn_Em_ and Mn_Em_ at room temperature. **f** Schematic illustration of the energy transfer mechanism in the Sn^2+^/Mn^2+^-co-doped Rb_4_CdCl_6_ system

The introduction of Mn^2+^ ions into Rb_4_CdCl_6_:*Sn*^2+^ results in an additional broadband emission component in the yellow–red region (denoted by as Mn_Em_). Figure 2a shows a pseudo-color map of PL and PL excitation (PLE) for a sample with an Sn^2+^ and Mn^2+^ doping ratio of 4.5% and 3.4%, respectively. The PLE distribution pattern for the two emission components is similar, indicating that the design of the energy transfer from [SnCl_6_]^4−^ to [MnCl_6_]^4−^ had been achieved successfully. Figure 2b displays the emission profiles under excitation at 300 nm. Notably, the profile of cyan emission component (485 nm) remains largely unchanged with the Mn^2+^co-doping, although its intensity decreases to 0.534 times that of the pristine emission in the Sn^2+^-doped Rb_4_CdCl_6_. Conversely, the extracted Mn_Em_ component (606 nm) exhibits a redshift of ~ 20 nm compared to the Mn^2+^-doped Rb_4_CdCl_6_ (Fig. S3). These results can be attributed to the formation of Sn–Mn pairs, which involve a short-range and strong interaction between Sn^2+^ and Mn^2+^ ions. As a result, the Sn^2+^ dopants can be classified into two species: Sn^2+^ ions with and without the adjacent Mn^2+^ ions.

### Analysis of Sn–Mn Dexter Energy Transfer Pair

As the PL excitation occurs in a fast process following the Franck–Condon approximation, from the ground state to the excited state, during which the surrounding lattice has no chance to reorganize, the difference in the local environment of Sn^2+^ can be reflected by changes in the excitation spectrum. The PLE profiles corresponding to the Sn_Em_ and Mn_Em_ components (denoted by Sn_Ex_ and Mn_Ex_, respectively) are carefully examined in Fig. 2d, e. In general, the PLE spectrum of n*s*^2^ ion-doped perovskite exhibits a fine molecule-like structure resembling that of a molecule that consists of specific excitation bands [47, 48]. Peak fitting analysis using Gaussian function was performed to identify the origin of each band in the PLE spectra [48]. The Sn_Ex_ and Mn_Ex_ exhibit similar A and C bands (^1^*S*_0_ → ^3^*P*_1_ and ^1^*S*_0_ → ^1^*P*_1_), but the bands in Mn_Ex_ have wider bandwidths compared to Sn_Ex_, as listed in Table S1. In addition, a B band (^1^*S*_0_ → ^3^*P*_2_), which is allowed by coupling with non-totally symmetric lattice vibrations, is observed in Mn_Ex_. This suggests that the Sn^2+^ ions adjacent to Mn^2+^ ions experience a relatively distorted lattice environment, which can be attributed to the smaller ion radii Mn^2+^.

The transient PL decay corresponding to Sn_Em_ and Mn_Em_ components was measured by excitation at C band (268 nm) at room temperature and is shown in Fig. 2e [48]. The decay curves of Sn_Em_ remained nearly unchanged with the co-doping of Mn^2+^, confirming that the Sn^2+^ specie that without adjacent Mn^2+^ ions maintained their pristine cyan STEs emission and were not significantly influenced by Mn^2+^ co-doping. In contrast, the lifetime of the Mn_Em_ decreased dramatically from 6.4 (Fig. S4) to 4.6 μs, indicating that the parity-forbidden property of the ^4^*T*_1_ to ^6^*A*_1_ transition had been removed, further confirming the strong interaction between adjacent Sn^2+^ and Mn^2+^ ions. Moreover, the decay curve of Mn_Em_ component can be well described by a biexponential fit, with two lifetime constants of 3.3 and 11.4 μs, accounting for 86% and 14% of the total decay, respectively. Notably, the dominant short lifetime constant is shorter than the lifetime of the Sn_Em_ component (3.5 μs), indicating the energy transfer occurs from FE rather than STE states of Sn^2+^ ions. Previous studies have shown that in the presence of STEs [14], once electrons and holes are photogenerated, they quickly become self-trapped, resulting in a more stable state with lattice distortion. However, in the presence of adjacent Mn^2+^ ions, a more stable state is provided, and the photogenerated electrons and holes move to the Mn^2+^ center corresponding to a short-range exchange-coupled interaction instead of becoming self-trapped, as illustrated in Fig. 2f.

**Fig. 3:**
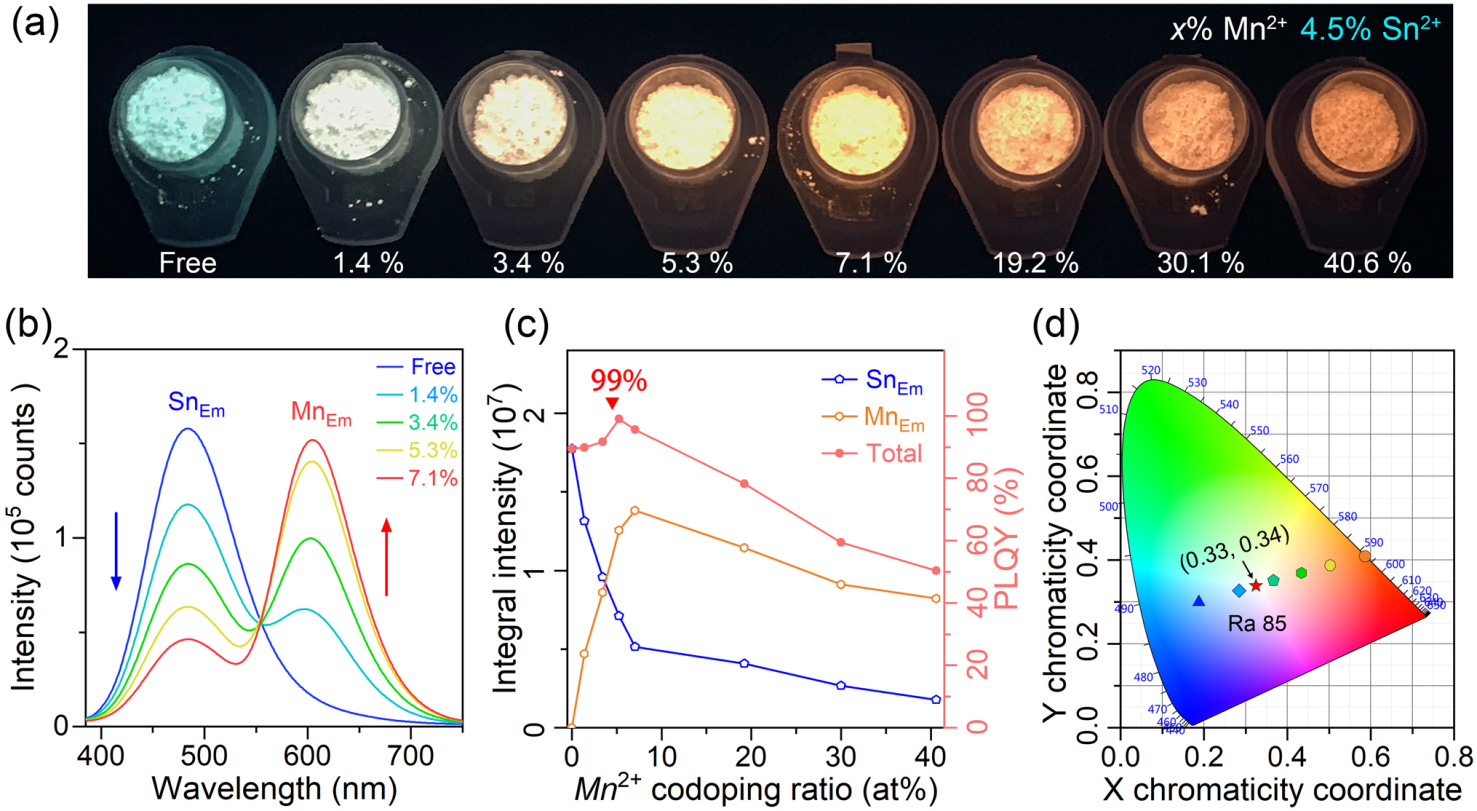
**a** Optical images of Sn^2+^/Mn^2+^-co-doped samples under UV light. **b** PL spectra of Sn^2+^/Mn^2+^-co-doped samples collected using an integral sphere. **c** Integrated intensities of Sn_Em_ and Mn_Em_ components of Sn^2+^/Mn^2+^-co-doped samples with different doping ratios, and PLQYs of the dual emissions. **d** Commission Internationale de l’Eclairage (CIE) chromaticity coordinates of the Sn^2+^/Mn^2+^-co-doped samples

In Fig. 3a, the optical image of as-prepared Sn^2+^/Mn^2+^-co-doped Rb_4_CdCl_6_ samples under UV light is shown, with a fixed doping rate of 4.5% for Sn^2+^ and varying from 0 to 40% for Mn^2+^. The PL color gradually transitions from cyan to white and then to orange as the Mn^2+^ co-doping ratio increases. The corresponding PL spectra, collected using an integral sphere, are shown in Figs. 3b and S5. The intensity of Mn_Em_ component increases with the Mn^2+^ doping ratio until it reaches a maximum intensity at a co-doping ratio of 7.1 at%, while the intensity of the cyan emission continuously decreases. Figure S6 demonstrates that the extracted Mn_Em_ component from different samples exhibits an identical profile at 606 nm, which is unaffected by the amount of Mn^2+^ co-doping. The integral intensities of dual emissions are presented in Fig. 3c. It is noteworthy that an appropriate amount of Mn^2+^ co-doping (< 7.1%) can improve the PLQY of the system from ~ 90% to near unity (> 99%) (Fig. S7). However, further increase in the Mn^2+^ doping amount leads to typical concentration-related quenching. To investigate the quenching processes, electron paramagnetic resonance (EPR) characterization was performed (Figs. S8 and S9). The EPR spectrum of Rb_4_CdCl_6_:*Mn*^2+^ with and without Sn^2+^ co-doping exhibited a similar six-hyperfine lines profile, indicating the long-range Mn–Mn dipolar interaction was not significantly influenced by Sn^2+^ ions [36]. The broadening of the six-hyperfine lines is observed with increasing the Mn^2+^ content, indicating a decrease in the average distance between Mn–Mn centers [36]. As the Mn^2+^ doping ratio increases, the short-range exchange-coupled Mn–Mn interaction becomes dominant, leading to a significant quenching in PL [36]. This quenching process can be attributed to the competition between Mn–Mn and Sn–Mn exchange coupling interactions. The near-unity PLQY suggests that internal energy transfer efficiency from Sn^2+^ to Mn^2+^ is a high (see details in Supporting information), prioritizing over the formation of STE and non-radiative vibrational relaxation since excited Sn^2+^ serves as the sole energy source of Mn_Em_. The combination of cyan Sn_Em_ and orange Mn_Em_ enables the creation of high-quality tunable cold/warm white light, where the relative intensity of the two components depends on the ratio of the two Sn^2+^ species, as follows:

1 $$\frac{{I}_\text{Cyan}}{{I}_\text{Orange}}=\frac{{c}_{\mathrm{Res}}}{{c}_{\mathrm{Pair}}}\times \frac{{QY}_{\mathrm{STE}}}{{QY}_{\mathrm{Mn}}}$$

where *c*_Res_ and *c*_Pair_ are the concentrations of the Sn^2+^ ion with and without adjacent Mn^2+^ ions and *QY*_*S*TE_ and *QY*_Mn_ are the PL efficiencies of the cyan STE emission and the Mn^2+^ *d-d* emission, respectively. When the doping concentrations of Sn^2+^ and Mn^2+^ ions are relatively low, the values of *QY*_*S*TE_ and *QY*_Mn_ are constant by approximation. Because short-range interactions occur between the Sn^2+^ and Mn^2+^ ions, Sn–Mn pairs arise when the Sn^2+^ and Mn^2+^ centers are less than a certain distance *r* apart. From a geometric crystallography perspective (Fig. S10), the fraction of the residual Sn^2+^ ions (without any adjacent Mn^2+^ ions) can be described as:

2 $${P}_{\mathrm{Res}}=\frac{{c}_{\mathrm{Res}}}{{c}_\text{Pair}+{c}_{Res}}=({1-Mn\mathrm{\%})}^{\mathrm{n}}$$

where *n* is the number of Cd^2+^ sites in a sphere with a Sn^2+^ ion as the center with a radius of *r*. According to Eqs. 1 and 2, the variation in the intensity of the Sn_Em_ component with Mn^2+^ content can be described as follows:

3 $${I}_{\mathrm{Res}}={I}_{0}\times ({1-Mn\mathrm{\%})}^{\mathrm{n}}$$

where* I*_0_ is the original intensity of the cyan broadband emission of Sn^2+^-doped Rb_4_CdCl_6_. The revolution of Sn_Em_ intensity after Mn^2+^ co-doping agrees well with the *n* = 14 relationship as shown in Fig. S11. The corresponding interaction range for the Sn^2+^ and Mn^2+^ ions is determined as ~ 0.9 nm, suggesting a Dexter energy transfer process. This finding further confirms the presence of exchange-coupled interaction in Sn–Mn pairs. As shown in Fig. 3d, The CIE coordinates of the PL shift from (0.19, 0.30) to (0.47, 0.38) as the Mn^2+^ concentration increases from 0 to 7.1%. The PLQY ranges from 90 to 99% and CCT ranges from 20,933 to 2,323 K. When the Mn^2+^ doping concentration is higher than 7.1%, the intensity of the Mn_Em_ component starts to decrease, but not as much as the Sn_Em_ component. As a result, the CCT of dual emission further decreased to 1,567 K. This coverage encompasses the variation range of sunlight. Notably, a CIE of (0.33, 0.34) with a CRI of up to 85 was achieved in the sample of ~ 3 Mn% and ~ 4.5 Sn% co-doped in Rb_4_CdCl_6_, which closely matches the standard white light required for illumination or display application.

**Fig. 4:**
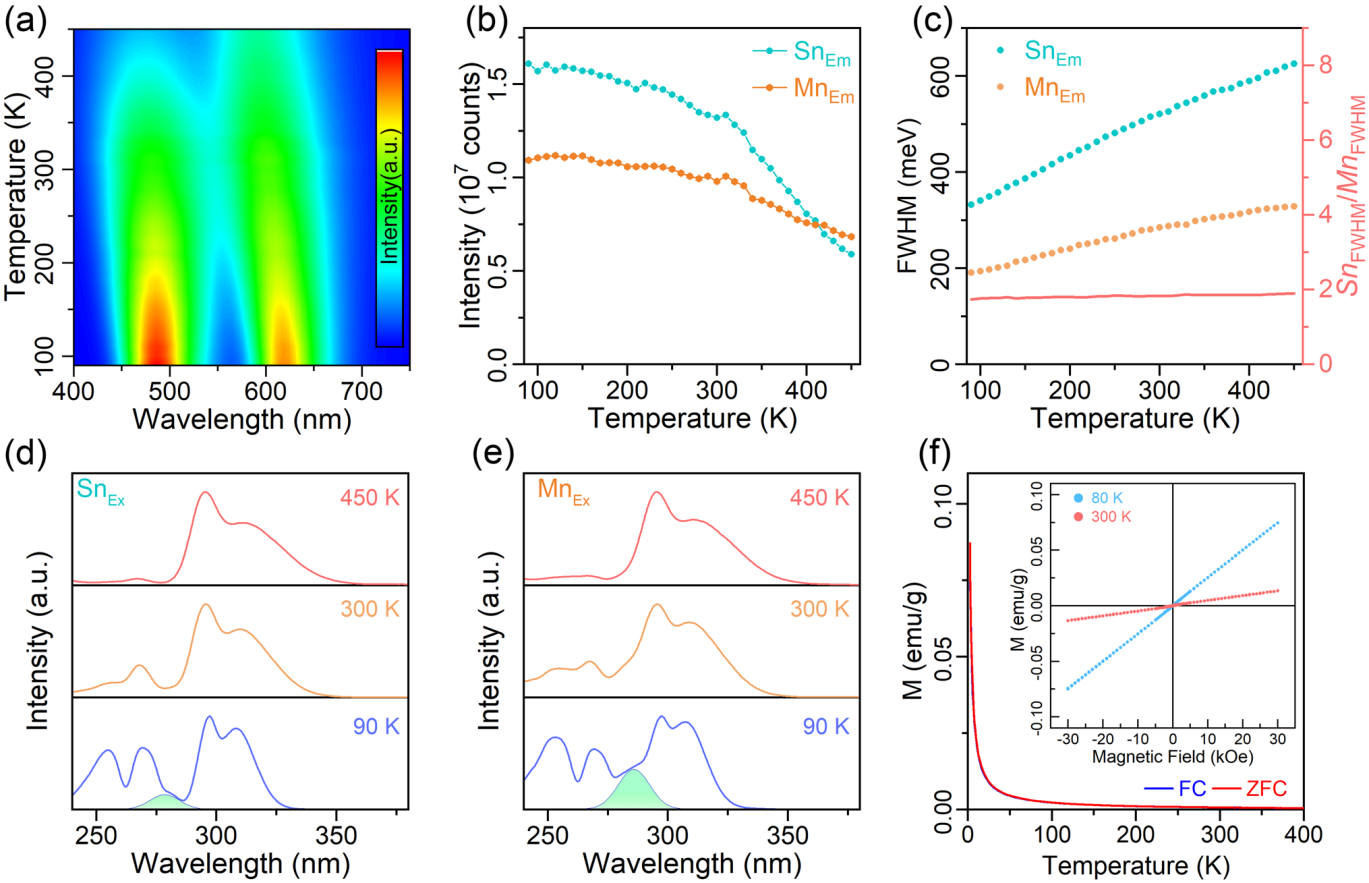
**a** Pseudo-color map of the temperature-dependent PL from the Sn^2+^/Mn^2+^-co-doped sample in the 90–450 K range. **b**, **c** Evolution of the integrated intensity and the FWHM with increasing temperature for Sn_Em_ and Mn_Em_ in the Sn^2+^/Mn^2+^-co-doped sample. **d**, **e** Excitation profiles corresponding to Sn_Em_ and Mn_Em_, respectively, at 90, 300, and 450 K. **f** FC and ZFC curves for the Sn^2+^/Mn^2+^-co-doped sample. The inset shows plot hysteresis loops obtained at 80 and 300 K

To further investigate the thermal stability of the dual emission and gain a deeper understanding of the highly efficient energy transfer from Sn^2+^ to adjacent Mn^2+^ in Rb_4_CdCl_6_ host, PL spectra were measured for Sn^2+^-doped and Sn^2+^/Mn^2+^-co-doped Rb_4_CdCl_6_ samples over a temperature range of 90–450 K. A pseudo-color map depicting temperature-dependent variations in PL intensity, wavelength, and FWHM is presented in Figs. 4a and S12. Both the Sn_Em_ and Mn_Em_ components exhibit a blueshift and broadening with increasing temperature. The Sn_Em_ component in the samples with and without Mn^2+^ doping shows a similar trend, providing further evidence for a short-range interaction between Sn^2+^ and Mn^2+^ ions. Based on this observation, the Mn_Em_ component was separately analyzed (Fig. S13). The integrated intensities of Sn_Em_ and Mn_Em_ as a function of temperature are shown in Fig. 4b. It is noteworthy noting that even at a temperature as high as 450 K, the Sn_Em_ and Mn_Em_ components retain 43.9% and 70.2% of their initial intensity at ambient conditions, indicating an excellent thermal stability. Conversely, as the temperature decrease to 90 K, a gradual increase in the intensity of Sn_Em_ and Mn_Em_ is observed, indicating the suppression of vibrational relaxation. These results suggest that the high energy transfer efficiency is not significantly influenced by the temperature. Figure 4b illustrates the narrowing of the Sn_Em_ and Mn_Em_ components as the temperature decreases. The relationship between the FWHM and the temperature can be described as follows:

4 $$\mathrm{FWHM}\left(T\right)=2.36\sqrt{S}\hslash {\omega }_{\mathrm{phonon}}\sqrt{\mathrm{coth}\frac{\hslash {\omega }_{\mathrm{phonon}}}{2{k}_{B}T}}$$

where *S* is the Huang–Rhys factor, ℏ*ω*_phonon_ is the phonon frequency, and *k*_B_ is the Boltzmann constant [3, 14]. The *S* factor is a parameter that provides an estimation of the electron–phonon interaction strength by measuring the average number of vibrational quanta involved in a PL process [49]. In the case of co-doped Sn^2+^ and Mn^2+^ ions in Rb_4_CdCl_6_ host, we assume the same phonon frequency for both emission components. By approximating the relationship between the *S* factors of Sn_Em_ and Mn_Em_ using the squared ratio of their FWHM, we find that the *S*_Sn_/*S*_Mn_ value is determined to be 3–3.6. This significant difference in the strength of the electron–phonon interaction between Sn_Em_ and Mn_Em_ could account for their distinct thermal stability.

Figure 4d and e depicts the PLE profiles of Sn_Em_ and Mn_Em_ at 90, 300, and 450 K, respectively. With increasing temperature, the PLE profiles of the two components gradually converge, as shown in Fig. S14, suggesting that the distortion caused by smaller Mn^2+^ radii becomes less significant. Interestingly, the PLE profile resembles like that of Sb^3+^-doped Cs_2_NaInCl_6_, which has a 3D-networked structure, at high temperature, while that resembles that of Sb^3+^-doped Rb_3_InCl_6_, which has a 0D-networked structure, at low temperature (Fig. S15) [50–52]. This temperature-dependent evolution of PLE indicates the relative intensity of A and C bands is independent on structure dimensionality. The similar temperature dependence of the PLE profiles for Sn_Em_ and Mn_Em_ further confirms that the energy transfer from Sn^2+^ to Mn^2+^ in the Sn–Mn pair occurs from the ground state rather than from the excited state [33]. To investigate whether the strong interaction between Mn^2+^ and Sn^2+^ is caused by the magnetic order of Mn^2+^, the magnetic properties of a sample co-doped with Mn^2+^ and Sn^2+^ (3.4% and 4.5%) were examined using temperature-dependent magnetization. As shown in Fig. 4f, the zero-field-cooled (ZFC) and field-cooled (FC) curves exhibit typical paramagnetic behavior, indicating disorder of the Mn magnetic moments in the Rb_4_CdCl_6_ host. The absence of magnetic ordering is further supported by the observed hysteresis at 80 and 350 K of 30 kOe, as shown in the inset in Fig. 4f.

### WLED Device Performance

**Fig. 5:**
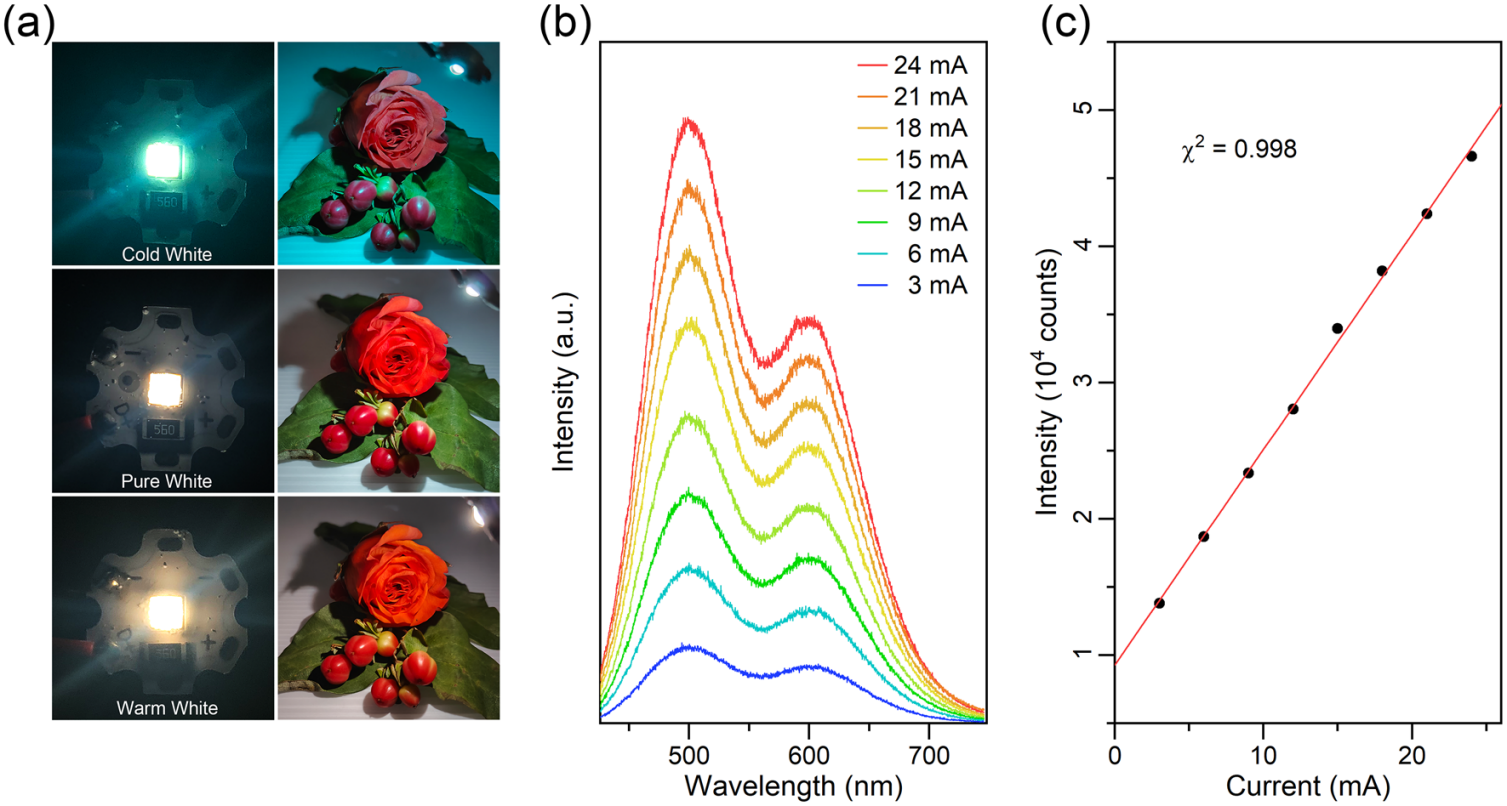
**a** Fabricated down-conversion cold/warm WLEDs and the color performances of flowers under illumination by the WLEDs. **b**, **c** Variations in the emission spectra and the intensity of a WLED with different driving currents

To demonstrate the potential application of Sn^2+^/Mn^2+^-co-doped Rb_4_CdCl_6_ as a single-component white emitter in lighting technology, phosphor-converting WLED devices were fabricated by directly placing the as-prepared sample powder onto a commercially available 295-nm UV chip. The fabricated WLED devices, ranging from cold to warm, are shown in Fig. 5a, along with corresponding photographs illuminated by these WLED devices. The photographs exhibit vivid and realistic colors, indicating excellent color discrimination. Figure 5b, c presents a series of emission spectra of WLED device at different currents, with the emission intensity increases monotonously with current. The normalized emission spectra, as shown in Fig. S16, exhibit the same profile, indicating that the CRI and CCT remain relatively unchanged at different currents. These results demonstrate the good photostability of white-light device, making it suitable for practical applications.

## Conclusions

In summary, our study successfully incorporated Sn^2+^ and Mn^2+^ ions into all-inorganic 0D Rb_4_CdCl_6_ perovskites, leading to the realization of near-unity and tunable dual-emission white light in a single material. The presence of short-range and strong interactions between adjacent Sn^2+^ and Mn^2+^ centers, facilitated by the formation of exchange-coupled Sn–Mn pairs, enabled highly efficient energy transfer process from Sn^2+^ as the donor to Mn^2+^ as the acceptor. The dual-emitting centers of Sn^2+^ and sensitized Mn^2+^ exhibited similar excitation profiles and could be easily adjusted by varying the Mn^2+^ doping content, allowing for precise control of the emission proportions and the generation of white light. A synchronous PL decay process observed in the dual-emission indicated stable dynamic color rendering, making it particularly advantageous for stroboscopic white-light applications. This research provides valuable insights for the design of high-performance single-component white phosphors and diodes, opening new possibilities for next-generation lighting technologies.

### Supplementary Information

Below is the link to the electronic supplementary material.

Supplementary file1 (PDF 1410 KB)
