# Supplementary material for: Achieving Tunable Cold/Warm White-Light Emission in a Single Perovskite Material with Near-Unity Photoluminescence Quantum Yield
Source: Nanomicro Lett. 2023 Aug 31;15:207. doi: 10.1007/s40820-023-01168-5 (PMC10471562; doi:10.1007/s40820-023-01168-5)
Supplement: Supplementary file 1 — Supplementary file1 (PDF 1410 KB) [file 40820_2023_1168_MOESM1_ESM.pdf]

Supporting Information for

Achieving Tunable Cold/Warm White Light Emission in a Single Perovskite Material with Near-Unity Photoluminescence Quantum Yield

Bo Zhou [1, 2, ‡], Aixuan Du[1, ‡], Dong Ding[2], Zexiang Liu[1], Ye Wang[3], Haizhe Zhong[1], Henan Li[4], Hanlin Hu[5, *], Yumeng Shi[4, *]

[1] International Collaborative Laboratory of 2D Materials for Optoelectronics Science and Technology of Ministry of Education, Institute of Microscale Optoelectronics, Shenzhen University, Shenzhen 518060, P. R. China

[2] Shandong Laboratory of Yantai Advanced Materials and Green Manufacturing, Yantai 264006, P. R. China

[3] Key Laboratory of Material Physics, Ministry of Education, School of Physics and Microelectronics, Zhengzhou University, Zhengzhou 450052, P. R. China

[4] School of Electronics and Information Engineering, Shenzhen University, Shenzhen 518060, P. R. China

[5] Hoffmann Institute of Advanced Materials, Shenzhen Polytechnic, Shenzhen 518060, P. R. China

‡ Bo Zhou and Aixuan Du contributed equally to this work.

*Corresponding authors. E-mail: hanlinhu@szpt.edu.cn (Hanlin Hu), yumeng.shi@szu.edu.cn (Yumeng Shi)

Supplementary Figures

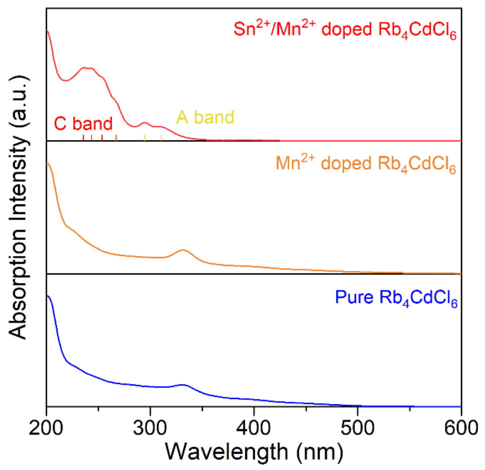

Fig. S1 UV-vis absorption spectra of pure Mn^{2+}-doped, Sn^{2+}/Mn^{2+}co-doped Rb_4CdCl_6

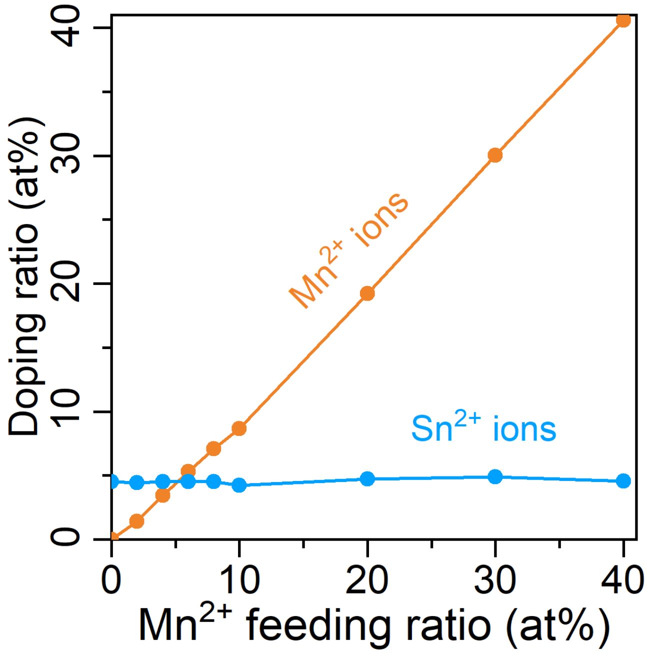

Fig. S2 ICP-OES results for variations in Mn^{2+} doping with different feed amounts. Sn^{2+} fixed at 5%

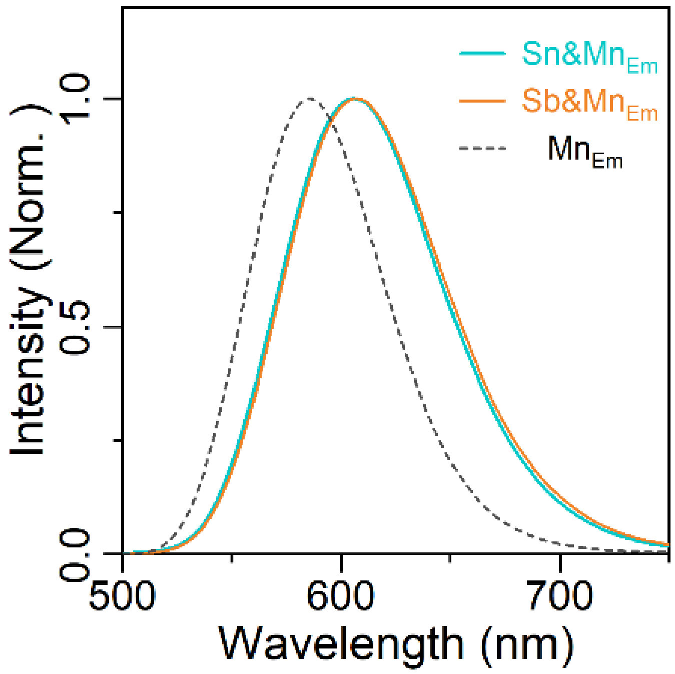

Fig. S3 The comparation of yellow emission from Mn^{2+} doped and Sn^{2+}/Mn^{2+} co-doped Rb_4CdCl_6. It is worth noting that Sn^{2+}/Mn^{2+} and Sb^{3+}/Mn^{2+} co-doped Rb_4CdCl_6 shows the similar yellow emission

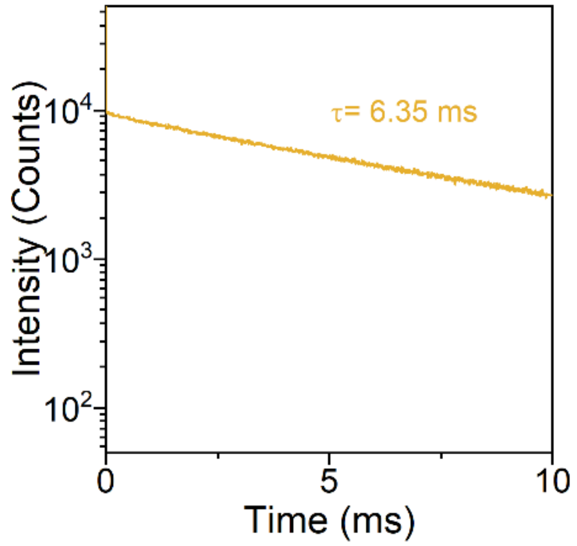

Fig. S4 Decay curve for Mn^{2+}-doped Rb_4CdCl_6 at room temperature

Interestingly, a typical 6.5 ms lifetime was observed for the Mn^{2+} doped Rb_4CdCl_6 sample. However, after Sn^{2+} co-doping a significant shortening of lifetime to 4.6 μs was observed together with the red shift of the PL peak of Mn^{2+} ions. This result indicates that Sb^{3+} has a strong effect on the emission of Mn. According to the previous reports, when Mn-based perovskites were scaled down to nanometer size, their lifetime is shortened from a few milliseconds to a few nanoseconds [S1]. The decrease of lifetime is attributed to the breaking of the forbidden transition $^4T_1$ to $^6A_1$. Therefore, in this system, the forbidden $^4T_1$ to $^6A_1$ transition may be broken by the effect of SOC or ns^2 electrons of the heavy nucleus Sn^{2+} ion. Further research is needed to reach deep understanding of the decay process.

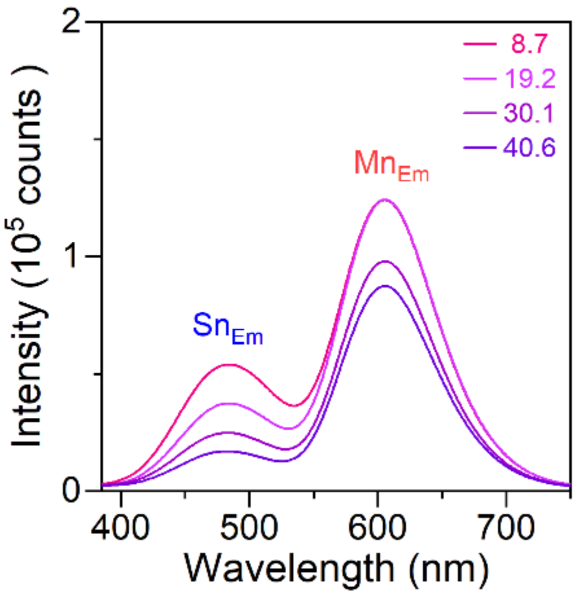

Fig. S5 PL spectra of Sn^{2+}/Mn^{2+} co-doped samples collected by an integral sphere

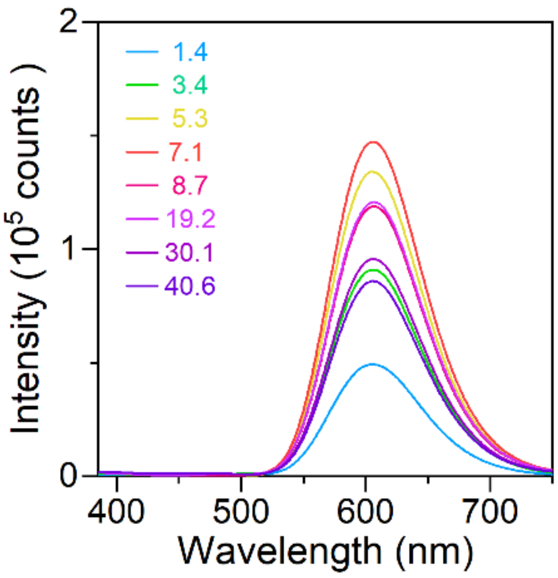

Fig. S6 Mn_{Em} component of Sn^{2+}/Mn^{2+} co-doped samples obtained by subtracting green self-trapped exciton emission from dual emission

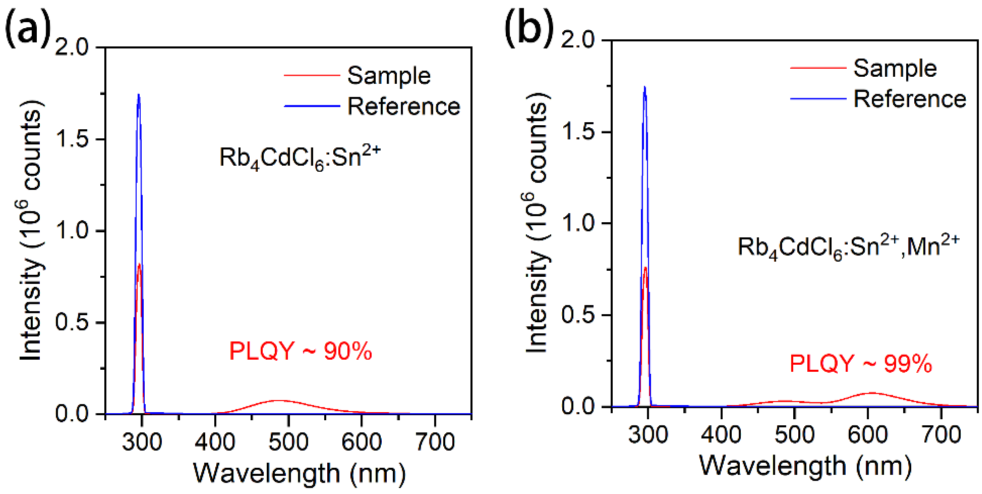

Fig. S7 PLQY of Sn^{2+} doped and Sn^{2+}/Mn^{2+} co-doped Rb_4CdCl_6 samples

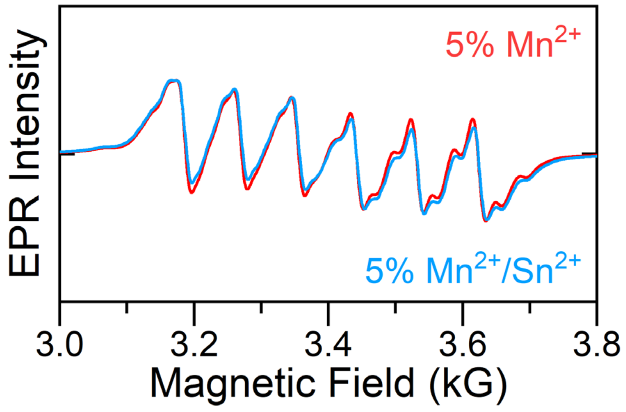

Fig. S8 Room temperature EPR spectra of 5% Mn^{2+} doped and 5% Mn^{2+}/Sn^{2+} co-doped Rb_4CdCl_6

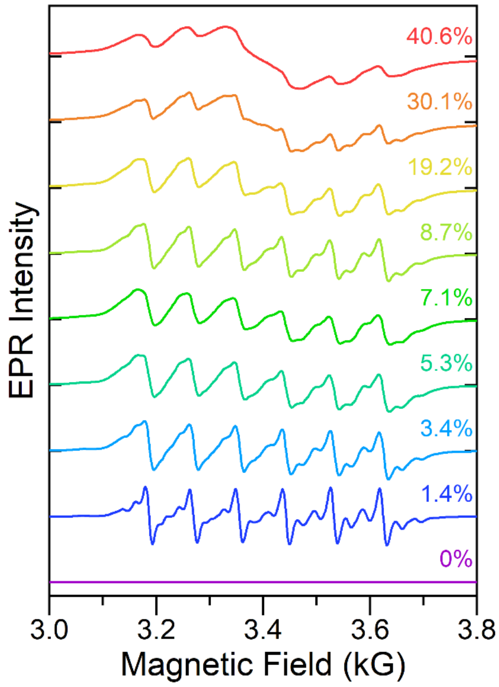

Fig. S9 Room temperature EPR spectra of Mn^{2+}codoped Rb_4CdCl_6: 5% Sn^{2+} from 0% to 40.6%. The spectra are offset for clarity

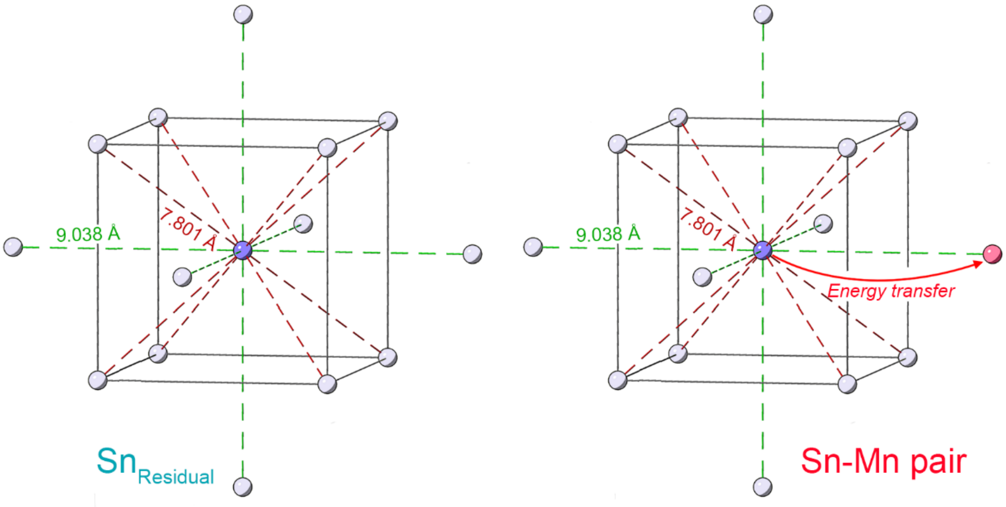

Fig. S10 Schematic diagram of the Sn^{2+}-Mn^{2+} pair in Rb_4CdCl_6 structure and the calculaiton of residual Sn^{2+}. Each Cd sites is surrounded by 8 first nearest and 6 second nearest Cd-sites. Rb^+ and Cl^- ions are hidden in the diagram

The derivation of this probability formula: Considering the very low doping of Sb^{3+}, assuming that there is only one site around Sb^{3+} for Mn^{2+}, the probability of Mn not occupying the site is (1-Mn%). In case of two sites, the value is (1-Mn%)(1-Mn%+δ%), where δ% delta represents the concentration change of Mn^{2+} caused by the doping of first Mn^{2+} ions. Considering a very small δ%, the formula can be simplified as $(1\text{-}Mn\%)^2$. Similarly, in case of n sites, the value is $(1\text{-}Mn\%)^n$.

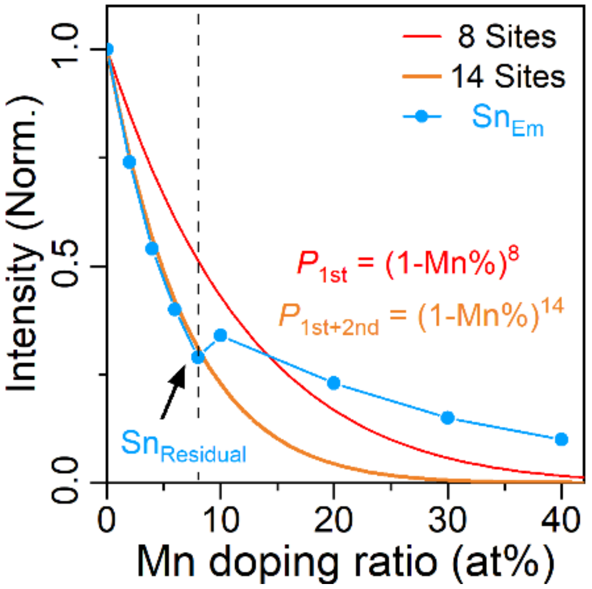

Fig. S11 The decrease of Sn_{Em} intensity agree well with the probability of Sn nearest and second-nearest sites not being occupied by Mn^{2+}

The EPR test results suggest that the Mn-Mn interaction gradually transitions from a relatively weak long-range interaction to a relatively strong short-range interaction with increasing Mn doping concentration. This suggests that as the concentration increases, adjacent Mn2+ and Mn2+ gradually become dominant. Similarly, the appearance of several Mn2+ adjacent to Sn2+ becomes significant. As a result, the competition between the Mn-Mn interaction and the Sn-Mn interaction may become significant, leading to a reduction in the energy transfer efficiency from Sn2+ to Mn2+. This may give a slightly higher intensity for cyan emission intensity for Sn than calculated.

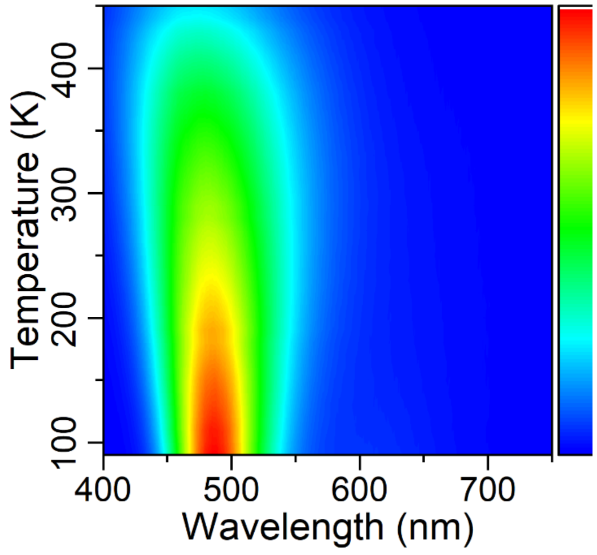

Fig. S12 Pseudo-color maps of temperature-dependent PL from Sn^{2+}/Mn^{2+} co-doped sample in range 90–450 K

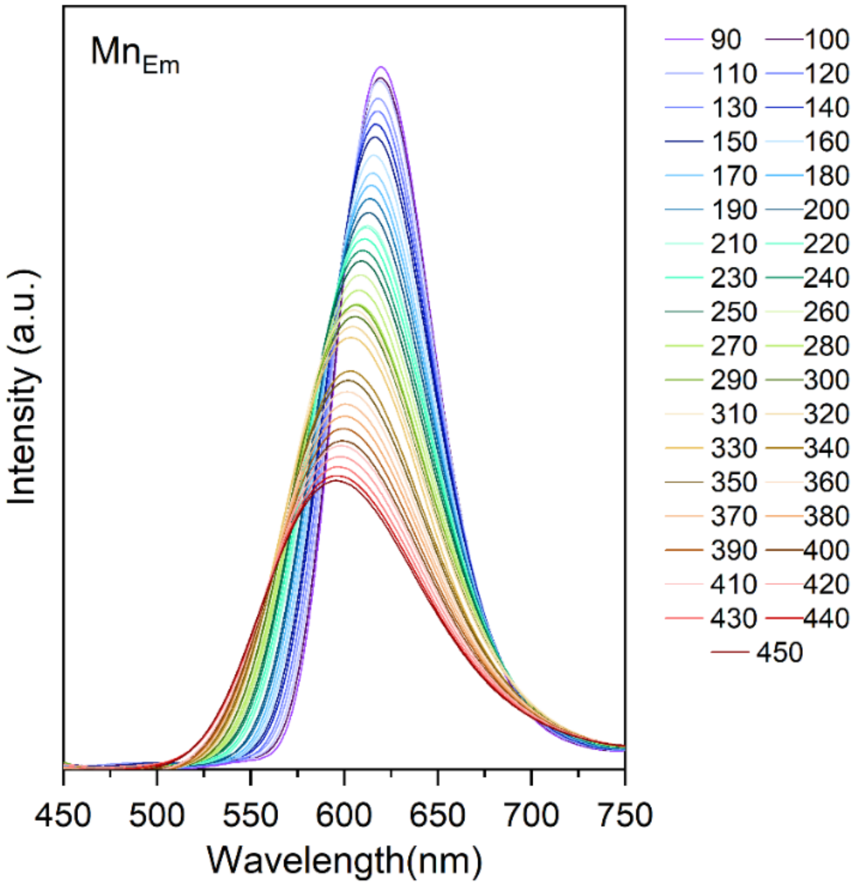

Fig. S13 Temperature-dependent PL of the Mn_{Em} component obtained by subtracting the cyan STE emission from the dual emission in the range 90 to 450 K

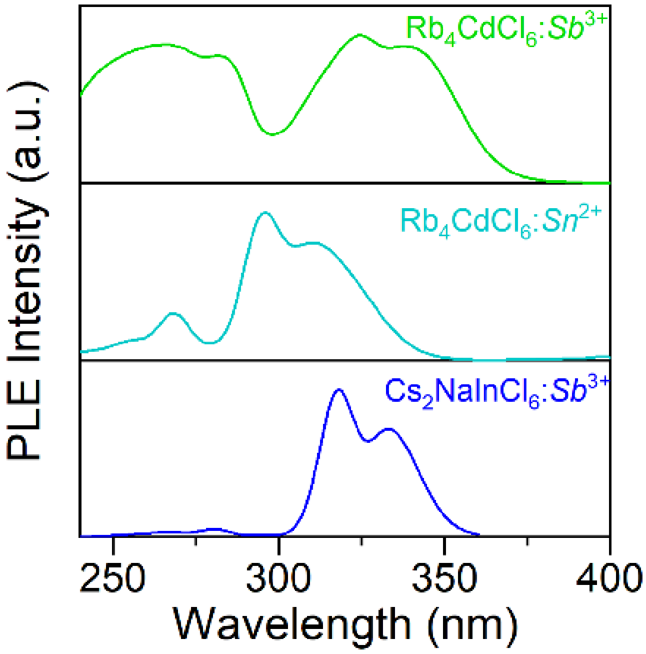

Fig. S14 PLE spectra of Sn^{2+} doped Rb_4CdCl_6 and Sb^{3+} doped $Cs_2NaInCl_6$ and Rb_4CdCl_6

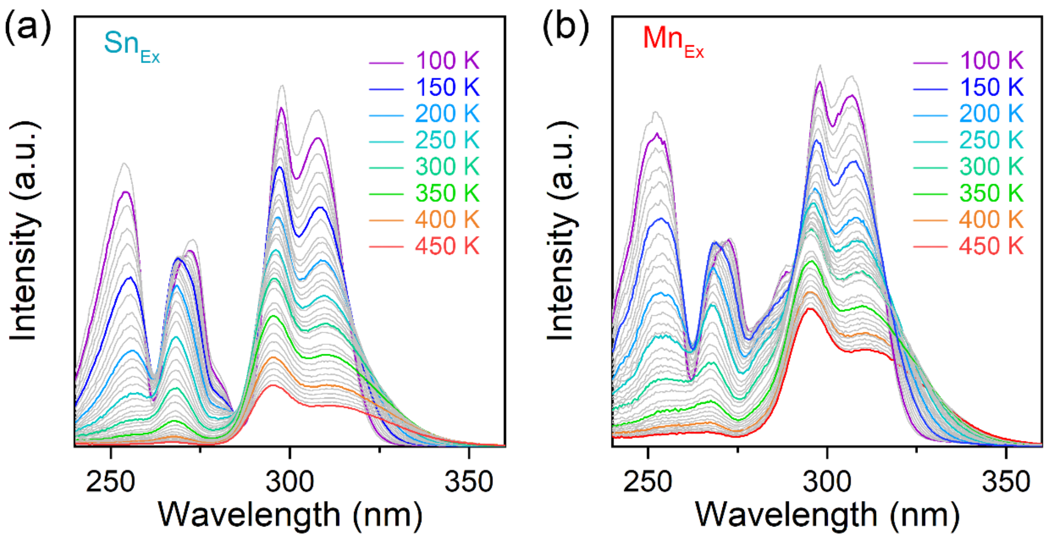

Fig. S15 Temperature dependent PLE spectra of Sn_{Em} (**a**) and Mn_{Em} (**b**) components

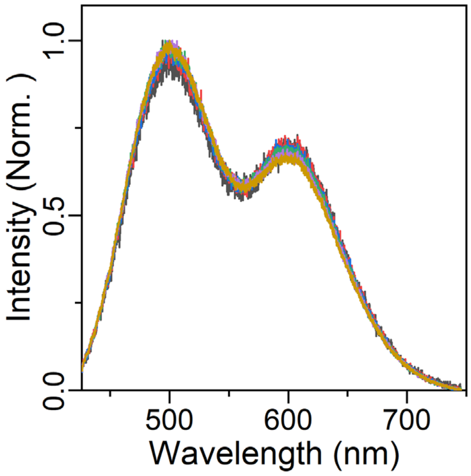

Fig. S16 Normalized emissions obtained from different currents show similar profile

Table S1 List of fitting result of A-, B- and C-bands from Sn_{Em} and Mn_{Em}

	**Sn_{Em}**		**Mn_{Em}**	
	**Center (nm)**	**FWHM (nm)**	**Center (nm)**	**FWHM (nm)**
**A1 band**	310.8	33.9	309.8	36.8
**A2 band**	294.3	11.3	294.1	11.4
**B band**	-	-	283	13
**C1 band**	268.3	12.1	267.3	13
**C2 band**	254.7	12.1	253.9	13
**C3 band**	242.5	12.1	241.4	13

Definition of internal energy transfer efficiency:

For short-range ET pairs, such as Sn-Mn pair, the "internal energy transfer efficiency" is defined as the ratio of the number of photons emitted by Mn^{2+} ions to the number of photons absorbed by adjacent Sn^{2+} ions. The "total energy transfer efficiency" is defined as the ratio of the number of photons emitted by Mn^{2+} ions to the number of photons absorbed by total Sn^{2+} ions. The "total energy transfer efficiency" can be obtained by averaging the "internal energy transfer efficiency" with the total Sn^{2+} ions.

Supplementary Reference

[S1] J. Almutlaq, W.J. Mir, L. Gutiérrez-Arzaluz, J. Yin, S. Vasylevskyi et al., Lead-free nanocrystals with high photoluminescence quantum yield and picosecond radiative lifetime. ACS Mater. Lett. **3**(3), 290–297 (2021). https://doi.org/10.1021/acsmaterialslett.0c00603
